# A health promotion perspective for the control and prevention of Brucellosis *(Brucella melitensis)*; Israel as a case study

**DOI:** 10.1371/journal.pntd.0010816

**Published:** 2022-09-26

**Authors:** Orna Baron-Epel, Samira Obeid, Dorit Kababya, Shiran Bord, Vicki Myers

**Affiliations:** 1 School of Public Health, Faculty of Social Welfare and Health Studies, University of Haifa, Mount Carmel, Israel; 2 North District Health Office, Ministry of Health, Israel; 3 Nursing Faculty, The Max Stern Yezreel Valley College, Yezreel Valley, Israel; 4 Ministry of Agriculture and Rural Development, Beit Dagan, Israel; 5 Health Systems Management Department, The Max Stern Yezreel Valley College, Yezreel Valley, Israel; Institute of Continuing Medical Education of Ioannina, GREECE

## Abstract

**Background:**

Brucellosis (*Brucella melitensis*) is endemic in many countries around the world, therefore, identifying what is required to control and prevent the disease is essential. The health promotion concept and five areas of action, presented in the Ottawa Charter (1986) may help understand how to go forward in the prevention of the disease. Israel serves as a case study.

**Aim:**

To identify barriers to the control and prevention of brucellosis (*Brucella melitensis*) in Israel by analyzing trends in incidence in conjunction with interventions implemented over the last seven decades, applying the health promotion areas of action.

**Methods:**

1. A document review approach was adopted to develop a list of interventions implemented in Israel to prevent and control brucellosis and identify barriers to implementation. These were analysed using the health promotion areas of action. 2. Data from the mandatory reporting of infectious diseases in Israel regarding brucellosis in humans between 1951 and 2021 are presented and analyzed in conjunction with the interventions implemented.

**Results:**

A large range of interventions were implemented following outbreaks of the disease. These interventions followed the health promotion areas of action, including mainly: policy, education and environment and brought about a decrease in the disease among both animals and humans. However, major interventions were discontinued after a few years. In addition, we identified some areas of action that could be much improved on. The interventions, in many cases were not simultaneously implemented or coordinated, decreasing the chances of them having the expected long term impact.

**Conclusions:**

Control and prevention of the disease in Israel is partial. Areas of action that could be improved include enforcement of regulations, strengthening community action and improving personal skills. Simultaneous and continuous implementation of the interventions may achieve the goals of sustained prevention and control. There seems to be a lack of a long-term strategy and an integrated holistic intervention approach that may contribute to the control and prevention of the disease.

## Author summary

Brucellosis, is a zoonotic disease endemic in the Middle East. Israel, can serve as a case study to try to identify barriers for prevention of the disease. In this study, we present the concept of health promotion and suggest it may help improve prevention and control of the disease. Using the health promotion areas of action (Building public policy, Creating supportive environments, Strengthening community action, Developing personal skills and Reorienting health services) we analysed the interventions implemented in Israel. We suggest that there are areas of action regarding the prevention of the disease that could be much improved on. In addition, interventions were not simultaneously implemented as needed, hindering success and there was a lack of a long-term strategy. For example, funding for prevention was discontinued after an initial decrease in incidence rates, enabling re-emergence of the disease. An integrated approach such as health promotion may provide the missing link to a successful holistic intervention that also includes the behavioural changes in human behaviour needed.

## Introduction

Brucellosis, a zoonotic disease endemic in the Middle East is also present in many other places around the world, including parts of China, Africa and the US [1,2]). It has been labelled as a re-emerging and neglected disease [1,3–5]. The disease is caused by the gram-negative coccobacillus *Brucella* that replicates within vacuoles of macrophages and other cells. The pathology of Brucella is divided into three phases: the incubation phase, the acute phase and the chronic phase that can eventually result in death [6]. More than ten species are known, of which two are the most important for humans and their livestock [2,7,8]. *B*. *melitensis* and *B*. *abortus* infect mainly small ruminants and cattle respectively and have a major economic effect as they reduce productivity of livestock [8,9] and infect humans [10]. Animals are infected by close contact with infected animals or consumption of products from infected animals. Animals should be vaccinated and kept away from infected animals or their products, with farm sanitation measures in place [2].

In humans, the disease can cause mild clinical signs but also more severe or chronic symptoms. The disease is transferred to humans through direct contact with tissues or blood from infected animals or consumption of contaminated animal products including unpasteurized milk and cheeses. Transfer through aerosol may also occur [2,6,10]. No human vaccine is available, although there are efforts to develop such vaccines [11], however, there are vaccines for animals [2]. To prevent infection, humans need to avoid contact with infected animals or their products, by adopting occupational and personal hygiene when coming in contact with animals including pasteurization of dairy products [2].

To tackle various zoonotic diseases, the One Health approach was developed [12,13], based on the concept of "holistic health", combining multiple disciplines to attain optimal health for people, animals, and the environment. Plumb et al. [14], suggested seven key factors for the prevention of brucellosis using the One Health approach: medicine, ecology, socioeconomics, policy, science, management and education. The disease operates within a complex web of interactions between animals, humans and the environment, making its prevention multifaceted.

Another concept proposed to tackle global efforts to improve health of humans, animals and environments is Ecohealth, suggested by Mi et al [15] to be a broader concept than One Health.

Building on the concepts of One Health and Ecohealth, we suggest adding the principles of health promotion to incorporate an emphasis on human behaviour [16].

### Health promotion as an additional approach to control brucellosis

The development of health promotion was led by the WHO as health and social professionals searched for a guide to build a healthier life for all. It is an action oriented field described as: "a social practice guided by principles and values such as participation and empowerment" [17]. The foundation of health promotion was provided by the Ottawa Charter in 1986 and included three strategies for action: [1] to enable, [2] to mediate and [3] to advocate. In addition, health promotion should be achieved through five areas of action: [1] building healthy public policy, [2] creating supportive environments, [3] strengthening community action, [4] developing personal skills and [5] reorienting health services [16]. Health Promotion, a multi-disciplinary field based on both philosophical democratic processes and community participation, emphasizes attention to the social and environmental determinants of health, for groups and populations [18–21]. A major part of health promotion is the centrality of human behaviour, where social processes influence behaviours and in turn, behaviours influence health [22–24].

### Brucellosis prevention and control by health promotion areas of action

Prevention and control of brucellosis can be divided into two distinct areas of intervention: the prevention and control of the disease in animals; and the prevention and control of the transfer of the disease to humans. A wide range of interventions have been proposed to contribute to the control and prevention of brucellosis [2,13,25–34] and can be categorized by the five Ottawa charter areas of action:

1. *Building healthy public policy* includes policies such as compulsory registration of all herds, national monitoring of disease prevalence, cooperation between involved ministries, implementation of testing and culling regulations and of immunization of animals at the most effective age, fair and fast compensation of farmers for animals lost to culling, and regulation of dairy production [30,31,35–41].
2. *Creating supportive environments* includes developing a strong local and national professional veterinary service, safe disposal of carcasses, use of disease-free animal feed and creating social norms that deter people from consuming non-pasteurized dairy products [37,41–45].
3. *Strengthening community action* includes collaboration between government and farmers and developing grass-roots organizations to advocate for prevention of the production, sale and consumption of non-pasteurized dairy products [37].
4. *Developing personal skills* includes education of farmers, dairy product manufacturers and the public regarding prevention and control of the disease. The educational goals should provide knowledge, training in skills for production of safe dairy products and should raise awareness of hazards [37,42,44,45].
5. *Reorienting health services* includes training of physicians and healthcare workers on how to identify, diagnose and treat at-risk patients [44].

All these interventions demand full cooperation between the healthcare service, the veterinary services, governmental organizations, community organizations and the media. Furthermore, interventions, which approach disease mitigation from different angles, should be implemented simultaneously to achieve control and prevention of the disease.

### Israel as a case study for the prevention and control of Brucellosis

As early as 1920 “sporadic cases of Malta fever” were reported in “Hygiene and disease in Palestine” by Masterman and the disease was reported to be clearly endemic in Palestine and all around the Mediterranean and the Middle East [46]. Initially both *Brucella melitensis* and *Brucella abortus* were reported, but by 1984–85 *Brucella abortus* was eradicated [35]. The success of the eradication of *Brucella abortus* was due to a successful intervention of testing for the disease and culling affected animals immediately, while simultaneously vaccinating female calves with a single dose vaccination [31,35]. Since then, in Israel, only *Brucella melitensis* has been responsible for outbreaks of the disease in both animals and humans. Most of the human cases are due to consumption of homemade unpasteurized dairy products in the Arab community [30,47].

Historically, the rural Arab population was an agricultural society that raised small ruminants for production of milk and meat, including home use. During the last century, most dairy cattle farmers in Israel were from the Jewish community while most farmers of sheep, goats and camels were from the Arab and Bedouin communities in the North and South of the country. The percent of Arabs and Jews living off agriculture has decreased over the years, however small ruminant herds are still mainly raised by Arab and Bedouin farmers and cattle mainly by Jewish farmers. Arab and Bedouin communities are at high risk of contracting the disease for two main reasons: contact with infected animals and consumption of dairy products manufactured from unpasteurized milk from affected animals. Within the Arab and Bedouin communities, animals are kept in close contact with humans in many of the villages, enhancing infection of humans. These behaviours are deeply embedded in the lifestyle and culture of some parts of the rural Arab community and therefore it is important to involve the community in developing pathways to prevent the disease [48,49].

Analysis of the interventions implemented in Israel, using the health promotion approach, can help identify gaps and challenges that persist in the control and prevention of brucellosis.

The aim of this study is to identify barriers to the control and prevention of brucellosis (*Brucella melitensis*) in Israel by analyzing the trend in incidence in conjunction with interventions implemented during the last seven decades, applying the health promotion areas of action.

Lessons can be learnt from the Israeli experience.

## Methods

### Ethical statement

The study does not include collection of personal data, all data is anonymized and we have permission from the University of Haifa ethics review board.

This study included two parts. The first part of the study is a qualitative document analysis of interventions intended to prevent and control brucellosis in Israel, using the five areas of action depicted in the WHO Ottawa Charter. First, a list of interventions implemented in Israel was identified by searching Google and Google Scholar using the words Israel, Brucellosis and intervention, both in English and Hebrew. In addition, reports not found on Google were provided by personal communication with Professor Arnon Shimshony (former director of the veterinary services at the Ministry Of Agriculture) and authors SO and DK. For identification of interventions we analysed 11 references, five in English and six in Hebrew. We also identified the existing regulations by which the ministries of Health and Agriculture act [41,50]. Barriers to implementation were identified within the references describing the control and prevention of brucellosis in Israel.

The interventions were matched with the appropriate health promotion area of action from the Ottawa Charter. The interventions were tabulated by area of action, and a short description of the intervention is given, with the year of implementation and by whom it was implemented. In addition, we present the degree of implementation reported, as far as possible with the available information. The analysis is presented in two tables, the first table includes the regulatory interventions (building healthy public policy) and the second table presents the other four areas of action, mainly the educational and organizational interventions.

In the second part of the study we present data regarding the incidence rates of brucellosis in humans for the years 1951 to 2021 obtained from the Ministry of Health reports on infectious diseases [39]. Human brucellosis in Israel has been notifiable by law since 1951. For 2020 and 2021, the data was available from the weekly epidemiological reports open to the public that included only the number of cases reported [39]. Rates were calculated for these years using the size of the population as reported by the Israel Bureau of Statistics.

**Fig 1:**
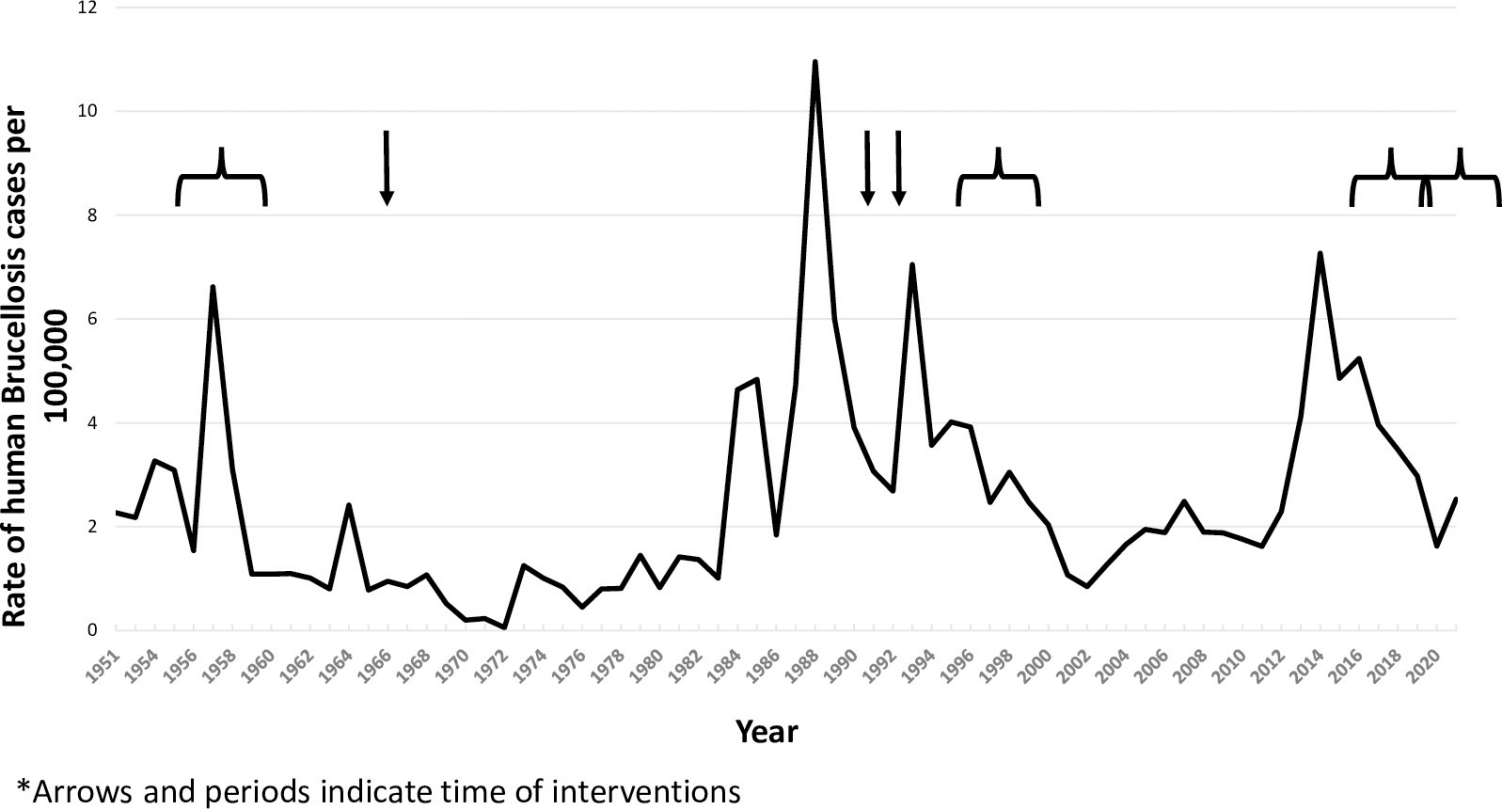
Reported human cases of brucellosis in Israel, rates per 100,000 and period of interventions, 1951–2021. *Arrows and periods indicate time of interventions

The years in which the major interventions to control and prevent brucellosis were implemented are indicated on Fig 1 showing the incidence of brucellosis in humans (Fig 1).

## Results

### An overview of the interventions implemented in Israel

In 1951, a report was published on prevention and control of the disease, describing diagnosis and vaccination of cattle and goats in Israel. It was reported that in 1949, a year after the declaration of the State of Israel, sporadic cases were diagnosed in cattle and small ruminants and serological tests were performed. The vaccinations, testing and culling were sporadic at best and not implemented in any methodical way [38].

Between 1952 and 1956 there was a reported increase in cases as herds were imported from Turkey and were apparently infected with the disease. The culling of most of these herds was not successful in the control and prevention of the disease [51] but did result in a major decrease in infected animals. In 1958, an immunological test survey was performed and many open herds (which periodically introduced new animals) tested positive for the disease but not the closed herds (which only bred from within the herd). At that time there was no funding to compensate the owners of sick animals that had to be culled [51]. Shimshony [36] suggested that eradication of the disease by culling infected flocks with full compensation would be economically unfeasible, and that whole flocks would be culled only at the farmer’s request. Herds not culled were branded and quarantined [36]. By 1964 the vaccine REV1 was adopted for use [51] and since 1980 only female animals up to the age of 6 months are vaccinated. During this period random blood sampling of herds was performed and infected herds were quarantined. The public was notified and advised not to buy cheese from non-regulated sources [36]. These steps were apparently partially successful and the disease was under control until 1985.

Two later major interventions were implemented as a result of increased incidence of the disease during the years 1984–1998 and between 2012 and 2018. These interventions included designated funding and an explicit implementation plan with goals. The regulatory basis for the monitoring, diagnosis and culling of the animals was based on the “1945 animal disease ordinance” and various parts were updated at different times. However, full implementation was not high or continuous, mostly due to lack of funding. Previous to the major intervention of 1994–98, sporadic actions were taken at the end of the 1980’s and at the beginning of the 1990’s [35]. Awareness of the problem increased at the beginning of the 90’s and by 1994 funding was provided to increase vaccination, testing and culling in order to decrease incidence of the disease. Furthermore, additional actions were taken including working with the population and raising awareness. For example, a local multidisciplinary coalition was formed in 1994 in an Arab town in the centre of Israel and an intervention including educational activities with the media, healthcare services, schools, women’s clubs and old age homes was implemented. At the same time the veterinary services worked with the farmers and traders to curb the disease. As Jaber et al. reported, there was a large decrease in human infections during the subsequent two years [42].

A formal national intervention due to the outbreak that started in 1984 was implemented during the years 1994–1998 by the veterinary services at the Ministry of Agriculture (MOA), in collaboration with the Ministry of Health (MOH) [31,35]. The intervention included developing and implementing the regulations regarding the treatment of herds, including reporting, testing, vaccinations and culling, alongside compensation. The aim was to test all small ruminant herds and keep them disease-free. The national intervention also included the training and instructing of farmers on handling of small ruminants. In addition, they worked with community leaders to advise on how to control and prevent the disease.

By 1996, this intervention was clearly having a positive effect, however the disease was not fully eradicated. The funding for the intervention was discontinued after this significant decrease in disease incidence, which led to the decision that there was no need for continued funding [52]. Unfortunately, the program was terminated too soon and by 2014 there was a new and strong outbreak.

Local interventions were implemented during 2014 by the MOH in a few towns in the north of Israel that had reported outbreaks of the disease. These interventions included collaboration with the local authority, healthcare services, the veterinary services and community leaders. Women were trained how to prepare cheese from pasteurized milk and how to bring about change in the community. Pamphlets and posters presenting control and prevention measures for brucellosis were developed and offered, and training was provided at schools. Similar interventions were implemented also in the southern part of Israel among the Bedouin communities and central Israel (personal communication with the authors SO and DK).

In the northern part of Israel during March 2015, the MOH developed and implemented a media campaign to raise awareness of the disease. This was aired on an Arabic language radio station. Twelve public announcements and numerous talk shows with professionals being interviewed were aired during the month [45].

During the years 2014–2017, the veterinary services of the MOA implemented an intervention labelled “long term program to decrease brucellosis in goat herds in the Negev 2014–2019”. This included registering herds, testing for the disease, culling and vaccinations. The program was discontinued in November 2017 for organizational reasons [43]. This program was implemented in the southern part of Israel and delivered mainly in areas that were found to be infected, and was later also implemented more widely in the rest of the villages [37].

In 2018, the State Comptroller and Ombudsman of Israel published their annual report regarding the activities of the veterinary services on brucellosis control and prevention [43]. They reported that the veterinary services failed to act on data showing increases in the rates of the disease. The intervention, they concluded, was not fully implemented, failed to vaccinate a high rate of herds and did not clear herds of sick animals. However, levels of brucellosis did decrease as can be seen in Fig 1.

A collaborative intervention program between the MOA, the Ministry of Environmental Protection and the National Parks and Nature Reserves was implemented during the period 2017–2021 in which 22 Bedouin students were trained to give talks in schools in the southern part of Israel. They instructed, trained and advised students, teachers and the community regarding prevention of brucellosis. The intervention aimed to increase knowledge and change attitudes towards brucellosis among Bedouins. In addition, a media campaign was run to increase awareness of the disease [44].

This intervention also included the collection of animal carcasses from the Bedouin communities during the years 2016–2018 [44]. The intervention continued till the end of 2021.

All these interventions included collaborations between governmental agencies and communities. For example, the MOH and the MOA organized several meetings for professionals from all areas to present and discuss the issues at stake both in the North and the South of the country. Most interventions were implemented in areas that exhibited high rates of the disease. However, consumption of non-pasteurized dairy products persisted in the Arab community both in the Southern and Northern regions [48] leaving the community vulnerable to the disease.

The interventions described are indicated on Fig 1 in conjunction with the trend of human brucellosis.

### Interventions to prevent and control brucellosis by the Ottawa Charter areas of action

The types of interventions performed in Israel (regulatory, educational and organizational) are presented in Tables 1 and 2 by the areas of action from the WHO Ottawa charter, period of intervention, description and degree of implementation.

**Table 1:** Regulatory interventions to control and prevent brucellosis in Israel.

Regulation	Year of implementation and implementing organization	Description of implementation	Degree of implementation
***Building healthy public policy*:**			
Farmers register their herds and animals [53]	Regulations for reporting animal diseases MOA*	Farmers register the herds with the veterinary services	Most herds are registered, however not all
Monitoring and reporting the prevalence of brucellosis in animals [31,38,41,54)]	Based on the 1945 animal disease ordinance. MOA	Reporting of clinical symptoms in animals	Partial at best
Monitoring the prevalence of brucellosis in humans [30,37,39]	The Public Health Ordinance 1940, brucellosis is a mandatory reported disease in Israel since 1951, MOH*	Compulsory reporting of infectious diseases including brucellosis	Regularly monitored by the MOH, may be underreported to some degree
Regulations for testing animals [31,36–38,41]	Based on the1945 animal disease ordinance, updated 1985, MOA (objectives: test 80% of herds)	1995-1998- large scale testing 2014–2018 increased serological tests	Large differences in levels of testing between the years
Regulations for culling of infected animals [31,35–37,40,41]	Based on the 1945 animal disease ordinance, updated 1985, MOA	Large scale interventions for culling of infected animals 1995–1998 2015–2017	Differences in levels of performance by years
Immunization and monitoring immunizations of animals [31,36–38,41]	A compulsory vaccine since 1989 implemented by the veterinary service, MOA. Compulsory reporting by farmers of litter aged 2–4 months old for vaccination, 4 times a year, since 1997	Large scale interventions for immunization of animals 1995–1998 2015–2017	Most herds are vaccinated but not all
Regulations for compensation of farmers [41]	Based on the 1945 animal disease ordinance, updated 2008, MOA	Farmers receive compensation for culling only if herd was registered with the MOA	The farmers complain of partial and delayed compensation
Regulation of production of dairy products [50]	By MOH Since the 50’s Updated 2011 and 2015	There are laws and regulations regulating the production of milk products	All commercial production and sales of milk products are regulated, however home production is difficult to regulate.

*MOA- Ministry of Agriculture, MOH Ministry of Health

**Table 2:** Educational and organizational interventions to reduce the burden of brucellosis in Israel.

Educational and organizational	Year of implementation	Description of implementation	Degree of implementation
***Creating supportive environments***			
Developing a strong local and national professional veterinary service [37,41,43]	Established in 1920	The veterinary services are a unit within the Ministry of Agriculture	Professional, employment and funding barriers to a strong and effective service
Hygiene of the animal-rearing environment [37,41,44]	2017-present	Regulations obliging farmers to dispose of carcasses in authorized places. In a pilot project the MOA funded the disposal in the south. In the rest of the country the local authorities and farmers are in charge of disposal	Partial cooperation of farmers. The pilot was partially successful. No sustainable enforcement.
Developing social norms that deter people from consuming non-pasteurized dairy products [42,44,45]	1994–5 2015–7	Working with religious leaders and local authorities. Community education Media campaigns	Mainly in communities with outbreaks
***Strengthening community action***			
Developing farmer organizations[37]	Farmers’ organization are in existence since the establishment of Israel, however include few Arab representatives	There are existing Israeli organizations that include Arab farmers, however, no organizations established and run exclusively by Arabs	Low representation of Arab farmers in the existing organizations
Grass roots consumer organization	Not reported	-	
***Developing personal skills***			
Training farmers [37,42,44]	Ongoing with increased activity in 1994–7 and 2017	Training of farmers on how to handle herds MOA	Ongoing mainly in localities with outbreaks
Training individual and private dairy product manufacturers [37,42,44]	Ongoing with increased activity in: 1990–1996 2014–8	Training women to prepare cheese from pasteurized milk in the north and south of the country by both the MOA and MOH	Ongoing training with difficulties reaching the relevant target groups such as individual women from the Arab community that prepare dairy products at home
Increasing the awareness and knowledge of communities and individuals [37,42,44,45]	1995–7 2015–2021	Information about the disease was distributed via pamphlets, schools, media, and health services. The target population included the generally Arab community and especially communities with reported outbreaks. Performed by both the MOA and MOH	25% of Arabs in the north of the country were aware of the disease from the media. The interventions were run mainly in towns with outbreaks of the disease
***Reorienting health services***			
Training physicians and healthcare workers [44]	1995–7 2015–6	Collaboration with healthcare services in towns with outbreaks	Sporadic. Collaboration with healthcare teams in towns with outbreaks

In general, Table 1 presents a large range of policies that can impact the control and prevention of brucellosis. However, it is clear from the reports and studies published that these policies were not consistently and fully implemented throughout the years [43,52]. They were not fully incorporated into the everyday work of those responsible for the implementation of the regulations. Furthermore, other activities that were implemented as part of large multifaceted interventions at specific times were not sustained (Table 2). Table 2 presents the other four areas of action. Again, interventions in all areas of action were implemented, however not always fully and persistently. More work should be done regarding “Creating supportive environments”, “Strengthening community action” and “Reorienting the healthcare services”. In these areas interventions were implemented but much more could be done to control and prevent brucellosis.

Interventions in all areas of action were implemented at one time or another during the years. However, the interventions were generally not implemented simultaneously all around the country or continuously, each stake-holder mostly implemented the interventions independently even though there were attempts to work together. For example, the media campaign in the north of the country was implemented in 2015, however at that time most of the Ministry of Agriculture’s efforts were focussed in the south of the country.

### Trends in Brucellosis in Israel

Fig 1 presents the incidence trend of the disease in humans in Israel since 1951 when reporting became compulsory. Three distinct periods of large outbreaks of *Brucellosis melitensis* in humans were documented. The first documented report covered the period 1950–1960, with the outbreak decreasing by the early 1960’s. The second outbreak began around 1984 and lasted for about 15 years. The third outbreak started in 2013 and leveled off towards the end of the decade. In between, the disease was never fully eradicated and remains endemic in Israel. In 2021 we observed an increase in the disease after about six years of continuous decrease.

## Discussion

The interventions implemented in Israel over the last 70 years brought about a decrease in the disease, both in humans and animals. However each time, following a few years of low incidence rates there was a subsequent increase in cases. When analysing the interventions by the areas of action of health promotion we can see that interventions were developed for each area of action. However, the interventions were not simultaneously implemented and the collaboration between the agencies in charge of the prevention and control of brucellosis was partial at best.

The main reason that emerged from analysing the reports and publications, is the fact that the interventions were not implemented in a consistent, continuous, long-term manner by the two ministries in charge of the control and prevention of brucellosis (Ministry of Health and Ministry of Agriculture). It seems that the periods of low incidence following interventions resulted in complacency, leading to the false notion that the interventions were successful and therefore decreased the motivation and willingness of professionals and decision makers to continue funding and running the interventions [43,52]. Both in 1997 and 20 years later in 2017 funding was discontinued for various reasons and as a result the vaccination, testing and culling of animals declined. Consequently, an increase in the incidence of the disease was to be expected as suggested by Shemesh and Yagupsky [52]. Therefore, we may say that the interventions according to the Ottawa charter were effective, however, the absence of a long-term maintenance strategy may be the problem. Furthermore, we suggest a lack of an organizational body that integrates all interventions and takes a holistic view of the activities for control and prevention. Coordination and concurrency of interventions are needed to achieve the goal. Cooperation between the various organizations and communities was partial and actions to strengthen communities to act on their own behalf have been insufficient.

The policies and regulations put in place throughout the years could have been sufficient for the control and prevention of brucellosis. Some were even put in place by the British prior to the establishment of the State of Israel. However, the enforcement of these policies and regulations was partial. These regulations are not only dependent on the activities of the MOA and their level of fundin but also depend on other players such as the farmers and the consumers of the milk products. For example, regulations demand that farmers register their herds with the veterinary service; however, enforcement by MOA is limited. This in turn, hinders vaccination of herds. The State Comptroller and Ombudsman of Israel in their report identified these obstacles and warned against them [43].

Human behaviour is an important player in the control and prevention of brucellosis. Developing personal skills is based on the idea that individuals should be provided with the information and skills to take control of their health, develop strategies to combat the disease, change norms so that the social environment promotes health and more. However, it seems that not enough has been done to change social norms regarding the consumption of non-pasteurized milk products [48] and improve the skills of individuals to lower their risk.

Discontinuation of the funding of the health education and communication interventions may have had an impact on the awareness of the disease and resulted in a reduction of positive health behaviours, resulting in an increase in consumption of non-pasteurized dairy products. Respondents in a survey in towns with brucellosis outbreaks reported higher levels of purchase and consumption of non-regulated dairy products compared to towns with no outbreaks [48]. In Baron-Epel’s study, knowledge about the disease was not associated with consumption of non-pasteurised dairy products. It seems that consumption of foods associated with traditions may be difficult to change. A long history of the tradition of consumption of cheeses from non-pasteurized goat and sheep milk, serve as a barrier to the control and prevention of the disease in humans. To achieve change, including changes in attitudes, social norms and behaviours, major social changes are needed, and these are difficult to bring about without collaboration with the community and long-term media and social campaigns. Although short-term communication interventions have been implemented they are always discontinued after a year or less, thus leaving the community to go back to its long lasting traditions. Health promotion concepts may help in obtaining these changes. It is important to emphasize that the regulated dairy products are available in all shops and are purchased in addition to the traditional unregulated cheeses.

The strategies suggested by the Ottawa charter for promoting health include cooperation and collaboration between the various organizations and communities. This was performed to a certain extent, but could be much improved, especially regarding the collaboration between the Ministries, the communities and their leaders. A recent study was performed where the community was asked to suggest how prevention and control of the disease could be achieved [49]. These ideas should be adopted and implemented and we await their implementation and evaluation.

Additional barriers exist to the success of the interventions. The fact that the Arab community in Israel is a minority within the Jewish majority may decrease the interest of the authorities in funding the interventions. In addition, the geopolitical situation in Israel could have a major effect on the barriers to the control and prevention of brucellosis, for example, herds belonging to nomadic herders (Bedouins) are not isolated from other herds, including transfer of animals from the Palestinian Authority in which brucellosis is also endemic. Farmers buy and sell animals across the border bringing in the disease. The disease can also infect a herd via wild animals from neighbouring countries and locally. Collaboration with Israel’s neighbours is not ideal to say the least, which serves as a barrier to successful control and prevention.

Lack of trust that this community has in the national services provided by the government is an additional barrier to disease prevention [48,55]. A lack of trust is especially influential in marginalized minority populations around the world. These minorities generally suffer from poverty and political exclusion and the roots of this distrust go much deeper, establishing a state of distrust from both directions. Overcoming this distrust is essential for the control and prevention of the disease; however, it depends on a wide range of factors, including economic and political factors that are beyond the scope of the organizations dealing with the prevention of brucellosis. One way to achieve this may be community participation in the development of the interventions implemented [49,56,57] and advocacy for the Bedouin community as suggested by the health promotion strategies.

It should be added that brucellosis is a disease that is not high on the health burden of disease list, with other health issues with greater burden of disease and health expenditures that call for more funding. This may also explain the limited investment in the interventions to decrease brucellosis.

This study has its limitations in depending on written documentation of interventions in Israel that we could find on the internet or from individual experts in the field. We may have missed additional documents not published. However, we do not think any major information regarding interventions is missing.

### Conclusions

Analysis of the interventions via the health promotion approach suggest the existence of interventions in all health promotion areas of action in Israel. However, control and prevention of the disease is partial at best. Areas of action that could be improved include enforcement of regulations, strengthening community action and improving personal skills. In addition, interventions should be simultaneously implemented by collaborating agencies. There seems to be a lack of a long-term strategy. Israel can serve as a case study for understanding the failures of countries to control and prevent the disease.
